# A trial of somatic gene targeting *in vivo *with an adenovirus vector

**DOI:** 10.1186/1479-0556-3-8

**Published:** 2005-10-12

**Authors:** Asami Ino, Yasuhiro Naito, Hiroyuki Mizuguchi, Naofumi Handa, Takao Hayakawa, Ichizo Kobayashi

**Affiliations:** 1 Department of Medical Genome Sciences, Graduate School of Frontier Science, University of Tokyo & Institute of Medical Science, University of Tokyo, 4-6-1 Shirokanedai, Minato-ku, Tokyo 108-8639, Japan; 2 Graduate Program in Biophysics and Biochemistry, Graduate School of Science the University of Tokyo; 3 Department of Environmental Information, Keio University, 5322 Endo, Fujisawa, Kanagawa 252-8520, Japan; 4 Laboratory of Gene Transfer and Regulation, National Institute of Biomedical Innovation, Asagi 7-6-8, Saito, Ibaraki, Osaka 567-0085, Japan; 5 Pharmaceuticals and Medical Devices Agency, Shin-Kasumigaseki Bldg. 3-3-2, Kasumigaseki, Chiyoda-ku, Tokyo 100-0013, Japan

## Abstract

**Background:**

Gene targeting *in vivo *provides a potentially powerful method for gene analysis and gene therapy. In order to sensitively detect and accurately measure designed sequence changes, we have used a transgenic mouse system, MutaMouse, which has been developed for detection of mutation *in vivo*. It carries bacteriophage lambda genome with *lacZ*^+ ^gene, whose change to *lacZ*-negative allele is detected after *in vitro *packaging into bacteriophage particles. We have also demonstrated that gene transfer with a replication-defective adenovirus vector can achieve efficient and accurate gene targeting *in vitro*.

**Methods:**

An 8 kb long DNA corresponding to the bacteriophage lambda transgene with one of two *lacZ*-negative single-base-pair-substitution mutant allele was inserted into a replication-defective adenovirus vector. This recombinant adenovirus was injected to the transgenic mice via tail-vein. Twenty-four hours later, genomic DNA was extracted from the liver tissue and the lambda::*lacZ *were recovered by *in vitro *packaging. The *lacZ*-negative phage was detected as a plaque former on agar with phenyl-beta-D-galactoside.

**Results:**

The mutant frequency of the *lacZ*-negative recombinant adenovirus injected mice was at the same level with the control mouse (~1/10000). Our further restriction analysis did not detect any designed recombinant.

**Conclusion:**

The frequency of gene targeting in the mouse liver by these recombinant adenoviruses was shown to be less than 1/20000 in our assay. However, these results will aid the development of a sensitive, reliable and PCR-independent assay for gene targeting *in vivo *mediated by virus vectors and other means.

## Background

Gene targeting, which is the precise alteration of genomic information by homologous recombination, has provided a powerful means of genetic analysis in microorganisms and mammalian systems [1]. In mouse systems, embryonic stem-cell lines modified *in vitro *can be used to generate mice that are altered at the germ-line level. If the gene targeting of somatic cells is made possible by gene transfer *in vivo*, it will facilitate the analysis of gene function, and provide a means of gene therapy for genetic and other diseases [2].

**Figure 1:**
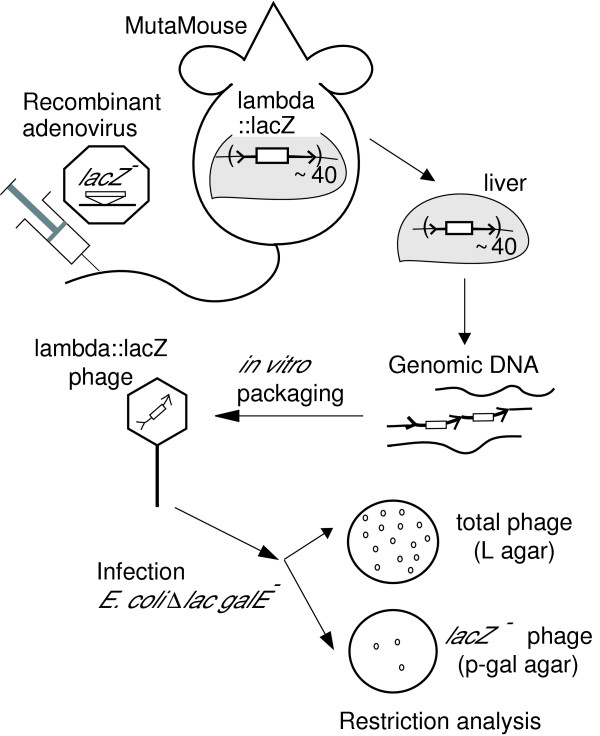
**Experimental steps to detect gene targeting *in vivo***. Gene targeting *in vivo *in liver cells was attempted after the delivery of donor DNA with an adenovirus vector. The gene with the required sequence change (*lacZ*^-^) on the lambda transgene in the mouse will be detected after its recovery in bacteriophage particles. Only *lacZ*-negative mutants can form plaques under the selective conditions.

There are two major inherent problems with the use of gene targeting *in vivo*. First, its low efficiency makes it difficult to detect and analyze. A sensitive and accurate measurement system is therefore needed to detect such low-frequency events. Although there have been several reports of gene targeting in the rat liver with specifically designed oligonucleotides [3,4], their reproducibility remains controversial [5]. PCR-based detection methods might thus be inaccurate and prone to various artifacts. In order to detect and measure gene targeting in mice with sufficient sensitivity, we used a bacteriophage transgenic-mouse system, MutaMouse, which has been developed for the detection of mutagenesis *in vivo *(Figure 1) [6]. The MutaMouse carries tandem repeats of the bacteriophage lambda genome with the *lacZ*^+ ^gene, in which the change to a *lacZ*-negative allele is detected after its *in vitro *packaging into viable bacteriophage particles.

The second major problem with gene targeting *in vivo *is that non-homologous recombination is much more frequent than homologous recombination in mammalian cells. Rare accurately modified cells are selected and purified in the case of embryonic stem cells that are treated *in vitro*. For gene targeting *in vivo*, imprecise modification would be detrimental for analytical uses and therapeutic purposes. Accurate gene modification has been achieved efficiently using replication-defective adenovirus vectors for gene delivery *in vitro *[7,8]. Fujita and colleagues used a mammalian plasmid as a model target [7]. The gene targeting was frequent (~10^-4 ^per cell) and analysis of the products revealed that homologous recombination was more frequent than non-homologous recombination. One possible reason for this high accuracy was protection of the viral DNA by the terminal protein, which is covalently attached to the ends of the viral DNA and to other viral proteins during its transfer to the nucleus and target DNA. Breaks in unprotected DNA would lead to non-homologous recombination.

The adenovirus is useful for gene delivery *in vivo *because it has a broad host-range, is easy to prepare to a high titer and only rarely integrates into the host genome by non-homologous recombination [9,10]. To date, more than 170 clinical studies have used recombinant adenovirus vectors to express cDNA in humans [11]. Numerous adenovirus-infection experiments have been carried out with mice, and have established that the injection of adenovirus recombinants into the mouse tail-vein leads to the expression of their genes in approximately one-half of the liver cells [12,13].

In the present study, we investigated gene targeting in the mouse liver using a replication-defective adenovirus vector and a transgenic mouse system (Figure 1). Although our initial attempts did not detect the predicted gene targeting (the frequency of the expected recombinants was less than 1/20,000 per lambda genome), the strategy and methods detailed here will aid the development of virus-mediated gene targeting *in vivo*.

## Materials and methods

### Bacteria, bacteriophages and plasmids

The bacteria, bacteriophages and plasmids used in this study are listed together with details of their construction in Additional file 1.

BIK12001 was used for the titration of bacteriophage lambda and the measurement of *lacZ*-negative bacteriophage lambda by phenyl beta-D-galactoside (p-gal) selection (see below). BIK1564 was used for the growth of all bacteriophage lambda strains in this study. BIK2206 was used for confirmation of the LacZ-negative phenotype of the bacteriophage selected with p-gal using 5-bromo-4-chloro-3-indlyl-beta-D-galactose (X-gal).

**Figure 2:**
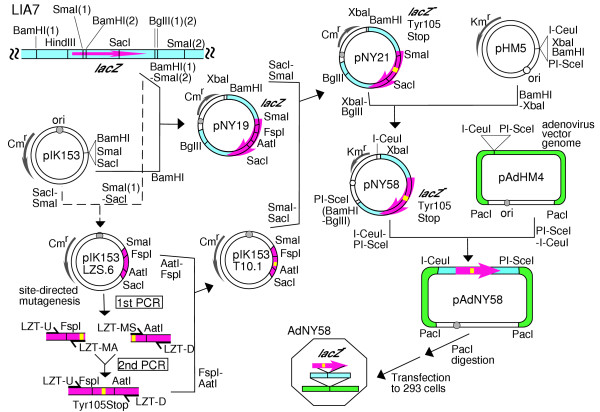
**Construction of the recombinant adenovirus AdNY58**. The bacteriophage lambda LIA7 was recovered from the MutaMouse by *in vitro *packaging. An SmaI-SacI fragment of LIA7 within its *lacZ *gene was inserted into pIK153. The Tyr105Stop mutation (Figure 3) was introduced into the resulting plasmid (pIK153LZS.6) using site-directed mutagenesis by PCR as follows. The PCR products generated with the primer pair LZT-U (5'-CGAAGAGGCCCGCAC-3') and LZT-MA (5'-TAATGGGCTAGGTTACGTTGGTGTAG-3'), and the primer pair LZT-MS (5'-TAACCTAGCCCATTACGGTCAATCC-3') and LZT-D (5'-GGCAACATGGAAATCGC-3') were mixed and used as templates for the second PCR with the primer pair LTZ-U and LZT-D. Replacement of an FspI-AatII fragment of pIK153LZS.6 by the FspI-AatII fragment of the resulting PCR product resulted in pIK153 T10.1. A BamHI-SmaI fragment covering the *lacZ *gene of LIA7 was inserted into the BamHI site of pIK153 (resulting in pNY19). pNY21 was made by replacing the smaller SmaI-SacI fragment of pNY19 with the homologous SmaI-SacI fragment of pIK153T10.1, which carries the mutant sequence. An XbaI-BglII fragment of pNY21 was used to replace the smaller XbaI-BamHI fragment of pHM5 (resulting in pNY58). pAdNY58 was made by replacement of the smaller I-CeuI-PI-SceI fragment of pAdHM4 with an I-CeuI-PI-SceI fragment of pNY58. The longer PacI fragment of pAdNY58 was transfected into 293 cells. The recombinant adenovirus AdNY58 was prepared and purified from the cell culture.

**Figure 3:**
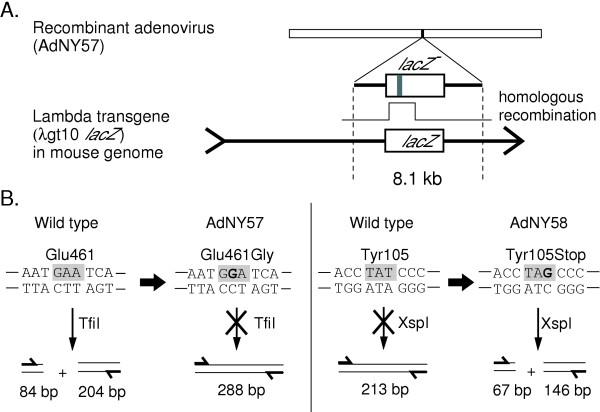
**Design for gene targeting and its detection**. (**A**) The donor carrying the mutant *lacZ *gene is inserted into an adenovirus vector. The *lacZ *mutation will be transferred to the *lacZ *gene of the lambda transgene in the mouse genome. (**B**) Expected sequence changes and their detection using restriction analysis.

The construction of the plasmids used in this study is detailed in additional file 1. The construction of pAdNY58 is also illustrated in Figure 2. The construction of pAdNY57 was as follows. The SmaI(1)-SacI fragment of LIA7 within the *lacZ *gene (Figure 2) was used to replace the shorter SmaI-SacI fragment of pUC18. The Glu461Gly mutation (Figure 3) was introduced into the resulting plasmid (pNY15) by site-directed mutagenesis using PCR [14] as follows. The PCR products generated with the primer pair LZG-U (5'-ACCGGCGATGAGCGAA-3') and LZG-MA (5'-GCCTGATCCATTCCCCAGCGACCA-3'), and the primer pair LZG-MS (5'-GGGAATGGATCAGGCCACGGCCGC-3') and LZG-D (5'-GGGCTGGTCTTCATCC-3'), were mixed and used as templates for the second round of PCR with the primer pair LZG-U and LZG-D. The MluI-BssHII fragment of the wild-type *lacZ *gene of pNY15 was replaced by the MluI-BssHII fragment of the PCR product. The targeted change in the resulting plasmid (pNY15G3.11) was confirmed by sequencing. pNY20 was produced by replacing the smaller SmaI-SacI fragment of pNY19 with the homologous SmaI-SacI fragment of pNY15G3.11, which carries the mutant sequence.

These two *lacZ *mutations were transferred back to lambda by homologous recombination *in vivo *[15] in order to generate LIA15 and LIA11, respectively. The recombinational transfer was carried out as follows. Cells of BIK12015 or BIK12018 were grown to OD_600 _= ~0.3 in LB (10 g bactotrypton, 5 g yeast extract and 10 g NaCl per liter) containing 20 μg/ml chloramphenicol, 0.2% maltose and 10 mM MgSO_4_. LIA7 was adsorbed onto the cells at a multiplicity of 1.0 at 37°C for 15 minutes. The mixture was shaken at 37°C until the OD_600 _dropped below 0.3. One drop of CHCl_3 _was added to the mixture, which was then shaken for 30 seconds. The mixture was centrifuged and the supernatant was recovered. The supernatant was assayed for BIK12001 on agar plates containing p-gal as detailed below. The plaques on the p-gal plates were isolated and analyzed for the designed sequence change by restriction of the PCR products (see *Analysis of the mutant bacteriophage DNA*).

### Selection of *lacZ*-negative bacteriophage with p-gal

The *lacZ*-negative bacteriophage particles were detected using positive selection [15,16]. BIK12001 cells were grown with shaking at 37°C to OD_600 _= 1.0 in LB containing ampicillin (50 μg/ml), kanamycin (20 μg/ml) and 0.2% maltose. The culture was centrifuged at 3,500 rpm for 15 minutes at 4°C. The pellets were dissolved into one-half the volume of LB containing 10 mM MgSO_4_. The bacteriophage was adsorbed onto these cells at room temperature for 20 minutes. To estimate the total number of bacteriophages, 2.5 ml molten 1/4 LB top agar (5 g LB broth base (Gibco BRL, Rockville, MD, USA), 6.4 g NaCl and 7.5 g Bactoagar per liter) was added to 0.25 ml of the mixture of cells and bacteriophages, and the entire content was poured onto a 1/4 LB plate (5 g LB broth base, 6.4 g NaCl and 15 g Bactoagar per liter). To estimate the number of *lacZ*-negative bacteriophages, 2 ml of the mixture of cells and bacteriophages, and 22 ml of molten 1/4 LB top agar containing 0.3% p-gal (Sigma Chemical Co., MO, USA), were mixed and poured onto four 1/4 LB plates. The plates were incubated at 37°C for 12 hours.

### Construction of recombinant adenoviruses

pNY56 was constructed by replacing the shorter XbaI-BamHI fragment of pHM5 by the XbaI-BglII fragment of pNY19 (Figure 2). pAdHM4 includes the entire genome of the recombinant adenovirus vector. The plasmid pAdNY56 was constructed by replacing the shorter I-CeuI-PI-SceI fragment of pAdHM4 by an I-CeuI-PI-SceI fragment of pNY56. The PacI fragment of pAdNY56 was transfected into cells of cell-line 293, which allows replication of the replication-defective adenoviruses. The recombinant adenovirus AdNY56 was prepared and purified as described previously [18]. Similarly, AdNY57 was constructed from pNY20 via pNY57 (Additional file 1), and AdNY58 was constructed from pNY21 via pNY58 (Figure 2, Additional file 1).

### Adenovirus infection

Female MutaMice (7 weeks old) were obtained from Covance Research Products Inc. (Denver, PA, USA). The MutaMice were maintained under specific pathogen-free conditions in the animal faculty of the Institute of Medical Science at the University of Tokyo, Japan. After the animals were anesthetized with Nembutal (Dainippon Pharmaceutical Co., Osaka, Japan), 3 × 10^9 ^plaque-forming units (PFU) of the recombinant adenovirus in 200 μl of PBS (137 mM NaCl, 8.10 mM Na_2_HPO_4_, 2.68 mM KCl, 1.47 mM KH_2_PO_4_, 0.9 mM CaCl_2_, 0.33 mM MgCl_2_) was injected into the tail-vein of each mouse using a 30-gauge needle. AdNY56 was injected into one mouse, AdNY57 was injected into two mice and AdNY58 was injected into two mice.

### Isolation of genomic DNA, recovery of lambda bacteriophage and measurement of mutant frequency

Twenty-four hours after injection, the mice were sacrificed. A lobe of the liver of each animal was excised, frozen by submersion in liquid nitrogen and stored in a 1.5-ml plastic tube at -80°C. Genomic DNA was isolated from the liver tissue with phenol-chloroform and precipitated by ethanol/sodium as described in the manual for MutaMouse. Lambda bacteriophage particles were recovered from the isolated DNA by incubation with packaging extracts (Mutaplax, Epicentre, WI, USA). The *lacZ*-negative mutants were detected by p-gal selection as described above. Each plaque on the selective agar was recovered in 100 μl of SM buffer (50 mM Tris-HCl (pH 7.5), 10 mM MgSO_4_, 100 mM NaCl and 0.01% gelatin). In order to verify the *lacZ*-negative phenotype, each isolate was assayed on agar with X-gal using a spot assay as follows. BIK2206 was grown in LB containing ampicillin (50 μg/ml) and tetracycline (10 μg/ml). Twice-concentrated culture (1.25 ml) was mixed with 6 ml molten LB/MM agar (100 ml LB medium, 0.75 g Bactoagar, 10 mM MgSO_4_, 0.2% maltose and 0.35 mg/ml X-gal) and spread on agar. A 10-μl aliquot of each bacteriophage sample was spotted onto these cells. The plates were incubated overnight at 37°C. The mutant frequency was estimated by dividing the number of PFU on the selective plate (as verified with X-gal) by the number of total PFU on 1/4 LB agar.

### Analysis of the mutant bacteriophage DNA

The *lacZ*-negative lambda bacteriophage DNA from the mice was analyzed using restriction enzymes following PCR. For the *lacZ*-negative lambda DNA from the AdNY57-treated mouse, PCR was carried out with the primer pair LG-1 (5'-TACCGGCGATGAGCGAAC-3') and LG-2 (5'-CTCCAGGTAGCGAAAGCC-3'). The 288-bp product was purified by ethanol/sodium precipitation, digested with TfiI (New England Biolabs, Beverly, MA, USA) (recognition site, 5'-G|AWTC-3' (W = A or T)) at 65°C and analyzed using agarose electrophoresis. The mutant sequence was resistant to TfiI, while the wild-type sequence was sensitive, yielding 204 and 84 bp fragments. The primer pair Lam-1 (5'-TACTGTCGTCGTCCCCTC-3') and Lam-2 (5'-CGCAGATGAAACGCCGAGT-3') was used for the *lacZ*-negative lambda DNA from the AdNY58-treated mouse. The 213-bp PCR product was digested with XspI (Takara Bio Inc., Shiga, Japan) (recognition site, 5'-C|TAG-3') at 37°C and analyzed using agarose electrophoresis. The wild-type sequence was resistant to XspI, while the mutant sequence was sensitive, yielding 146 and 67 bp fragments.

## Results

### Experimental design for the detection of gene targeting *in vivo*

Figure 1 illustrates our experimental design for the sensitive detection of gene targeting *in vivo*. The MutaMouse carries approximately 40 copies of bacteriophage lambda gt10*lacZ *on a chromosome [6,19]. The single integration site is located in band C on chromosome 3 [20]. Our target sequence was the wild-type *lacZ *gene. The donor DNA was delivered to the liver cell nuclei by tail-vein injection of the recombinant adenovirus. Genomic DNA was isolated from the liver and its *in vitro *packaging allowed the recovery of the lambda genome in viable bacteriophage particles. A *lacZ*-negative mutant bacteriophage was selected as a plaque-former in an *Escherichia coli *mutant defective in the *galE *gene on an agar plate containing p-gal. This chemical is converted by the *lacZ *gene product (beta-galactosidase) into UDP-galactose, which accumulates in the absence of the GalE protein to induce cell death. The ratio of the mutant plaque-formers to the total plaque-formers was used to estimate the fraction of the mutated gene. The mutant gene was further analyzed using restriction enzymes.

Replication-defective recombinant adenoviruses constructed by an *in vitro*-ligation method were used to deliver the donor DNA [18,21]. Figure 3 shows the structure of the recombinant adenoviruses used in the present study (see Figure 2, Additional file 1, and Materials and methods for further details). An 8077-bp fragment of lambda gt10*lacZ *was inserted into the E1 deletion site of the mutant adenovirus [18,21]. AdNY56 had wild-type *lacZ*, while AdNY57 and AdNY58 had a point mutation in *lacZ *(Figure 3B).

AdNY57 was constructed so as to introduce a point mutation at the active site of LacZ. The target sequence was the 5' GAA that codes for Glu461, which is essential for the activity of LacZ [22,23]. AdNY57 was expected to change its second base (that is, the 1437 th base) from A to G, thereby generating the Glu461Gly mutant, which shows a 76-fold decrease in activity [23]. The mutant and wild-type sequences can be distinguished using the restriction enzyme TfiI (Figure 3B).

AdNY58 was constructed so as to introduce a point mutation at the 5' TAT that codes for Tyr105. AdNY58 was expected to change its third base (that is, the 369th base) from T to G, thereby generating the Tyr105Stop mutant. The mutant and wild-type sequences can be distinguished using the restriction enzyme XspI (Figure 3B).

### Control experiments

We demonstrated that *lacZ *mutants that were predicted to be generated by the recombinant adenovirus could be selected with p-gal as follows. Bacteriophage lambda strains carrying the mutations were produced by transferring each mutation on a plasmid back to lambda through homologous recombination in *E. coli *(as detailed in Materials and methods). The two bacteriophage strains, lambda gt10*lacZ*^- ^Tyr105Stop (LIA11) and lambda gt10*lacZ*^- ^Glu461Gly (LIA15), were then used in the p-gal selection. As shown in Table 1, lambda with wild-type *lacZ *showed a plaque-formation efficiency of less than 1/10,000 on the selective agar relative to that on the non-selective agar. By contrast, each of the mutant lambda strains showed similar or slightly decreased plaque-formation efficiency on the selective agar. We concluded that the expected targeted product with AdNY57 and AdNY58, if it was produced, should be selected and measured using the p-gal-selection procedure.

**Table 1:** Selection efficiency of lambda *lacZ*-negative mutants

**Lambda**	**Genotype**	**Titer**	**Titer on p-gal selective plate**	**Relative plaque formation**
LIA7	*lacZ*^+^	2.2 × 10^10^	1.9 × 10^6^	8.6 × 10^-5^
LIA11	*lacZ*^- ^(Tyr105Stop)	9.6 × 10^10^	8.8 × 10^10^	9.2 × 10^-1^
LIA15	*lacZ*^- ^(Glu461Gly)	1.4 × 10^10^	1.3 × 10^10^	9.1 × 10^-1^

### Delivery of donor DNA and measurement of mutant frequency

The recombinant adenovirus particles (3 × 10^9 ^PFU in 200 μl of PBS) were injected into the tail-vein of a MutaMouse. It is well established that the adenovirus genome accumulates in the liver cell nuclei after tail-vein injection [12,13]. Most of the hepatocyte nuclei are expected to receive several copies of the adenovirus genome under these conditions (see Discussion). After 24 hours, the liver was excised from the MutaMouse, genomic DNA was isolated from the liver tissue and the lambda genome was recovered as a bacteriophage particle by *in vitro *packaging. The *lacZ*-negative phage was detected selectively on agar with p-gal. The plaques on these selective plates were isolated and the LacZ-negative phenotype was confirmed on agar plates containing X-gal. The mutant frequency was estimated as the fraction of the *lacZ*-negative phage (Table 2). The control mouse (animal number 0) received no injections.

**Table 2:** Detection of *lacZ*^- ^phage

Packaging exp.	RAd	Genotype	Animal number	Packaging	Total number of plaque formers	*lacZ*^- ^plaques	Mutant Frequency	Expected genotype
1	None	Not relevant	#0	Tube 1	4.5 × 10^4^	4	8.9 × 10^-5^	n.t.
				Tube 2	3.2 × 10^4^	3	9.4 × 10^-5^	n.t.
				Tube 3	8.5 × 10^4^	8	9.4 × 10^-5^	n.t.
				
							average 9.2 × 10^-5^	
	
	AdNY56	*lacZ*^+^	#1	Tube 4	8.5 × 10^4^	8	9.4 × 10^-5^	n.t.
				Tube 5	6.4 × 10^4^	3	4.7 × 10^-5^	n.t.
				Tube 6	8.8 × 10^4^	1	1.1 × 10^-5^	n.t.
				
							average 5.1 × 10^-5^	

2	None	Not relevant	#0	Tube 7	4.9 × 10^4^	5	10 × 10^-5^	n.t.
				Tube 8	6.6 × 10^4^	4	6.1 × 10^-5^	n.t.
				Tube 9	5.1 × 10^4^	10	20 × 10^-5^	n.t.
				
							average 12 × 10^-5^	
	
	AdNY57	*lacZ*^- ^(Glu461Gly)	#2	Tube 10	3.8 × 10^4^	6	16 × 10^-5^	0/6
				Tube 11	3.0 × 10^4^	7	23 × 10^-5^	0/7
				Tube 12	4.5 × 10^4^	9	20 × 10^-5^	0/9
				
							average 20 × 10^-5^	total 0/22

3	None	Not relevant	#0	Tube 13	3.7 × 10^4^	6	16 × 10^-5^	n.t.
				Tube 14	6.0 × 10^4^	5	8.3 × 10^-5^	n.t.
				Tube 15	4.4 × 10^4^	4	9.1 × 10^-5^	n.t.
				
							average 11 × 10^-5^	
	
	AdNY57	*lacZ*^- ^(Glu461Gly)	#2	Tube 16	1.3 × 10^4^	9	69 × 10^-5^	0/9
				Tube 17	3.9 × 10^4^	19	49 × 10^-5^	0/19
				Tube 18	6.5 × 10^4^	26	40 × 10^-5^	0/26
				
							average 53 × 10^-5^	total 0/54

4	None	Not relevant	#0	Tube 19	2.6 × 10^5^	8	8.5 × 10^-5^	n.t.
	
	AdNY57	*lacZ*^- ^(Glu461Gly)	#3	Tube 20	1.6 × 10^5^	5	6.3 × 10^-5^	0/5
				Tube 21	4.1 × 10^5^	9	8.6 × 10^-5^	0/9
				
							average 1.5 × 10^-5^	total 0/14

5	None	Not relevant	#0	Tube 22	3.3 × 10^4^	3	9.1 × 10^-5^	n.t.
	
	AdNY58	*lacZ*^- ^(Tyr105 Stop)	#4	Tube 23	8.6 × 10^4^	4	4.7 × 10^-5^	0/4
				Tube 24	3.1 × 10^4^	3	9.7 × 10^-5^	0/3
				
							average 7.2 × 10^-5^	total 0/7

RAd: Recombinant adenovirus

n.t.: Not tested.

The mutant frequencies of the AdNY56-injected and control mice were similar (Table 2, Experiment 1), and did not differ significantly from those reported previously using this method (see [15] and the references cited therein). No significant increase in the mutant form of the gene was induced by injection of the recombinant adenovirus: the mutant frequency of the AdNY57- and AdNY58-injected mice was similar to that of the control mouse, which was approximately 1/10,000 (Table 2).

**Figure 4:**
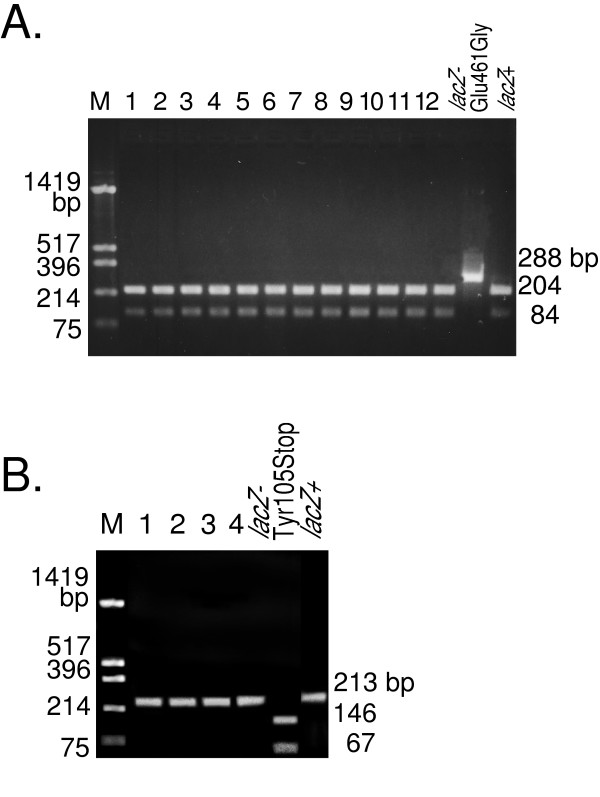
**Restriction analysis of the *lacZ*-negative gene from mice treated with a recombinant adenovirus**. (**A**) AdNY57-injected mouse. The PCR product of the lambda bacteriophage DNA with primers that flank the target site is 288 bp long. The wild-type PCR product is cut with TfiI into 84 and 204 bp fragments, whereas the Glu461Ala mutant PCR product is not cut. Lane M: Marker DNA prepared by HinfI digestion of the plasmid pUC19; 1–12, *lacZ*-negative bacteriophages from animal number 2; *lacZ*^+^: Lambda bacteriophage recovered from control mouse; *lacZ*^-^Glu461Gly: lambda bacteriophage LIA15. (**B**) AdNY58-injected mouse. The PCR product of the lambda bacteriophage DNA with primers that flank the target site is 213 bp long. The Tyr105Stop mutant PCR product is cut with XspI into 146 and 67 bp fragments, whereas the wild-type product is not. Lane M: Marker DNA prepared by HinfI digestion of plasmid pUC19; 1–4, *lacZ*-negative bacteriophages from animal number 3; *lacZ*^+^: Lambda bacteriophage recovered from control mouse; *lacZ*^-^Tyr105Stop: lambda bacteriophage LIA11.

All of the *lacZ*-negative bacteriophages were purified and their *lacZ *genes were analyzed using restriction-enzyme treatment of the PCR products (Figure 4). As shown in Figures 3B and 4A, the PCR product of the Glu461Gly mutant, as predicted from the AdNY57 injection, could not be cut with TfiI. By contrast, the wild-type and most of the other possible mutants could be cut with TfiI. In fact, all of the *lacZ*-negative bacteriophages from the AdNY57-injected mouse were cleavable with this restriction enzyme. As shown in Figure 3B and 4B, the PCR product of the Tyr105Stop mutant, as predicted from the AdNY58 injection, could be cut with XspI. By contrast, the wild-type and most of the other mutants could not be cut with XspI. None of the *lacZ*-negative bacteriophages from the AdNY58-injected mice were cleavable with this restriction enzyme.

We did not detect the expected gene replacement in any of the isolates. Moreover, the gene-correction frequency by these adenovirus constructs was shown to be less than 1/20,000 in the present system.

## Discussion

Here we attempted to perform gene targeting in a transgenic mouse system that allowed the sensitive detection of mutagenesis by various agents, such as those directly interacting with DNA in the liver and other organs [24,25]. The limit of sensitivity in this system was 1/20,000 (see also [15]). This procedure might provide an alternative to the PCR-based assay for gene targeting *in vivo*, although our initial trials did not detect any of the expected recombinants.

In the present system, the sensitivity appeared to be limited by the high level of spontaneous mutagenesis in the target gene. The MutaMouse system was produced to detect mutagenesis at numerous sites within a gene, rather than to study gene targeting. Experimental designs involving the specific selection of homologous recombination events, such as those used in the previous work *in vitro *[7], would therefore be preferred.

Also, in the present system, a successful gene-targeting event would not be distinguishable in the phenotype of the mouse cell. In transgenic mice with a single copy of the mutant *lacZ *gene [26], correction to the wild-type gene would result in a direct positive readout in the mouse body (for example, through staining with dye). However, as the authors admit, it would be difficult to detect the targeting events with a high sensitivity. The presence of multiple copies of the target gene would improve the sensitivity because the *lacZ*^+ ^allele is dominant over, and epistatic to, the *lacZ*^- ^alleles with respect to the above phenotype. The MutaMouse carries multiple (approximately 40) copies of the target gene, which amount to 0.4% of the genome. This should be able to improve the sensitivity of detection of gene targeting, although the sensitivity is limited by spontaneous mutagenesis. In addition, the presence of tandem repeats might have other types of negative effect on gene targeting, as detailed below.

How efficient is adenovirus infection and delivery to the hepatocyte nucleus? Tail-vein injection is an established method for the delivery of adenovirus to liver cells. The average copy number of a replication-defective recombinant adenovirus genome per liver cell has been estimated as 14–28 copies using Southern hybridization after tail-vein injection of 5 × 10^9 ^PFU of the virus [12]. This corresponds to 40% of the injected adenovirus. Fluorescence *in situ *hybridization revealed that, after tail-vein injection of 2 × 10^9 ^PFU, all of the hepatocyte nuclei had 1–100 copies of a recombinant adenovirus genome, with an average of 20 copies [27]. After tail-vein injection of 2 × 10^8 ^PFU of a recombinant adenovirus with the *lacZ *expression cassette, 40% of the hepatocytes expressed beta-galactosidase [13]. We assumed that the majority of the liver cells received several copies of the adenovirus genome, at least sufficient for gene expression, after injecting 3 × 10^9 ^PFU in our experiment. (We cannot raise the titer any more because of the toxicity of the virus.) This type of information can be confirmed by Southern hybridization and fluorescence *in situ *hybridization.

The gene-targeting frequency with recombinant adenoviruses *in vitro *varies from ~10^-7^–10^-4 ^per cell [7,8,28]. We did not detect any signal using recombinant adenovirus for gene delivery in the mouse liver. In order to achieve gene targeting *in vivo *using an adenovirus vector or any other means, it will be necessary to increase the frequency of gene targeting. So how can we achieve this goal?

The efficiency of gene targeting *in vitro *varies from one locus to another [29,30]. Such locus-dependence might reflect drastic effects of the chromatin structure on the frequency of homologous recombination [30,31]. Thus, the target transgene could be placed at a different locus that is known to be a hot spot in gene targeting in embryonic stem (ES) cells.

Repetitive sequences are methylated in the mouse genome [32]. Ikehata and colleagues suggested that the whole coding region of the MutaMouse *lacZ *transgene is methylated to a high degree at every CpG site [33]. One possible reason for this phenomenon is that the CpG content of the *lacZ *gene (9%) [34] is much higher than the average CpG content of the mouse genome (~1%) [35]. Methyl-CpG binding protein 2 (MeCP2) might bind to methylated CpG and somehow compact chromatin [36]. Furthermore, Manuelidis analyzed the structure of a mouse chromosome bearing a huge (~11 Mb) insert of a tandem-repeated transgene (~1,000 copies) [37]. This transgene was localized on an arm of chromosome 3 at a distance from the centromere. According to Manuelidis, the transgene is heterochromatic and highly condensed. Therefore, the MutaMouse transgene might be heterochromatic. The accessibility of nucleases to the heterochromatic structure is lower than that of euchromatin [38,39]. Reducing the copy number of the transgene and/or using another transgene that is lower in CpG content might increase gene targeting, although the decrease in copy number might affect the sensitivity of detection. An important experiment that can be done is to test whether the coding region of the MutaMouse lacZ transgene is really heterochromatic, using, for example, CHIP assay with the antibody against the methylated histones and PCR primers on the lacZ genes.

Chromosome replication is known to stimulate homologous recombination. Partial hepatectomies in mice might stimulate liver cell proliferation and DNA replication, which in turn might stimulate recombination. Hara et al. (1999) reported that partial hepatectomies increased mutagenesis with *N*-ethyl-*N*-nitrosourea, which is a direct-acting DNA-ethylation agent, in the MutaMouse [40].

It might be easier to modify the donor DNA than the recipient DNA. One can generate recombinogenic damage on the donor DNA. Irradiating adenovirus particles with ultraviolet light of 1500 J/m^2 ^resulted in an approximately three-fold increase in their mutual homologous recombination [41]. Recombinogenic cross-links are induced by some mutagens, such as psoralens, cisplatin (*cis*-diamminedichloroplatinum) and mitomycin C [42]. Such agents, both mutagenic and recombinogenic, might be suitable for gene targeting *in vivo *if they are shown to be active in mutagenesis in a transgenic-reporter mouse system. The effect of such recombinogenic damage might be much larger with replication-defective adenovirus recombinants than with replication-competent adenoviruses, because their replication-intermediates are responsible for their high recombination frequency [41,43-45].

The gene-targeting frequency is strongly dependent on the length of homology; the frequency increases as the homology length increases up to 10 kb [46-48]. If the deviation from this rule above 10 kb is due to the shearing and/or degradation of longer DNA after electroporation in embryonic stem cells, donor DNAs that are protected by the DNA binding proteins in the adenovirus particle might show greater length dependence over a wider range of values. Adenoviral vectors with a larger capacity for inserts, which are known as high-capacity 'gutless' vectors [49-51] might therefore be suitable for use in this approach.

## Conclusion

Here we attempted to perform gene targeting in a transgenic mouse system that allowed the sensitive detection of mutagenesis. The frequency of gene targeting in the mouse liver by these recombinant adenoviruses was shown to be less than 1/20000 with the sensitive and PCR-independent detection system.

## List of abbreviations

PCR, polymerase chain reaction; PFU, plaque-forming unit; RFLP, restriction fragment length polymorphism; p-gal, phenyl-beta-D-galactoside; X-gal, 5-bromo-4-chloro-3-indlyl-beta-D-galactose

## Competing interests

The author(s) declare that they have no competing interests.

## Authors' contributions

AI carried out the injection of the recombinant adenovirus and the analysis of the mouse DNA. YN and HM constructed the recombinant adenovirus. NH injected the recombinant adenovirus to the mouse. YN constructed the experimental design as well as cloning of the part of lambda DNA from the MutaMouse genomic DNA. IK provided the original experimental idea and coordinated the experimental design. All authors read and approved the final manuscript.

## Supplementary Material

Additional file 1 Bacterial strains, plasmids, bacteriophage strains and recombinant adenovirus constructs. Click here for file
