# Supplementary material for: A trial of somatic gene targeting in vivo with an adenovirus vector
Source: Genet Vaccines Ther. 2005 Oct 12;3:8. doi: 10.1186/1479-0556-3-8 (PMC1277836; doi:10.1186/1479-0556-3-8)
Supplement: Additional file 1 — Bacterial strains, plasmids, bacteriophage strains and recombinant adenovirus constructs. [file 1479-0556-3-8-S1.DOC]

**Additional file 1. Bacterial strains, plasmids,** bacteriophage strains and recombinant adenovirus constructs

| Strain | Properties | Comments |
| --- | --- | --- |
| Bacteria (*Escherichia coli*) | |  |
| BIK12001 | Strain C; deletion *lac, galE*^-^, *kan*, *bla* | [15] |
| BIK2206 | Strain C; deletion *lac*, *tet*, *bla* | [15] |
| BIK1564 | Strain K-12; MC1061; Δ(*codB*-*lacI*) 3 | Laboratory collection [52] |
| BIK806 | Strain K-12; *recD*::Tn*10* derivative of AB1157 | [53] |
| BIK12015 | BIK806 [pNY21] | This work |
| BIK12018 | BIK806 [pNY20] | This work |
| Bacteriophage lambda | |  |
| λgt10 | *λb527, srlλ3º, imm434, srlλ4º, srlλ5º* | Not used in the present work/ [54] |
| LIA7 | λgt10 *imm434*::*lacZ* | Recovered from Mutamouse/ [6] |
| LIA11 | λgt10 *imm434*::*lacZ*^-^, Tyr105Stop (TAT to TAG) | This work |
| LIA15 | λgt10 *imm434*::*lacZ*^-^, Glu461Gly (GAA to GGA) | This work |
| Plasmids |  |  |
| pIK153 | p15A origin, cml gene and a multiple cloning site from pUC119 | Figure 2. [55]. |
| pUC18 | *bla*, pMB1(mutant) origin | Laboratory collection. [56] |
| pIK153LZS.6 | *cml*, part of lacZ gene | Figure 2. |
| pIK153T10.1 | *cml*, part of lacZ^-^ gene, Tyr105Stop (TAT to TAG) | Figure 2. |
| pNY15 | *bla*, part of lacZ gene | SmaI(1)-SacI fragment of LIA7 (Figure 1) was inserted into pUC18 |
| pNY15G3.11 | *bla*, part of lacZ^-^ gene Glu461Gly (GAA to GGA) | MluI-BssHII fragment of PCR product (that carries the mutant sequence) was inserted into pNY15 |
| pNY17 | *cml, lacZ*^+^ | [15] |
| pNY19 | *cml, lacZ*^+^ | Figure 2. [15]. |
| pNY20 | *cml, lacZ*^-^, Glu461Gly (GAA to GGA) | SmaI-SacI fragment of pNY15G3.11 (that carries the mutant sequence) was inserted into pNY19. |
| pNY21 | *cml*, *lacZ*^-^, Tyr105Stop (TAT to TAG) | Figure 2. |
| pHM5 | pMB1(mutant) origin, *kan*, pUC18-derived multiple cloning site between I-CeuI and PI-SceI sites | Figure 2. A plasmid for adenovirus vector construction [21]. |
| pNY56 | *kan*, *lacZ*^+^ | XbaI-BglII fragment of pNY19 (that carries part of λgt10 *lacZ*) (Figure 1) was inserted into pHM5 |
| pNY57 | *kan*, *lacZ*^-^, Glu461Gly (GAA to GGA) | XbaI-BglII fragment of pNY20 (that carries part of λgt10 *lacZ*) was inserted into pHM5 |
| pNY58 | *kan*, *lacZ*^-^, Tyr105Stop (TAT to TAG) | Figure 2. XbaI-BglII fragment of pNY21 (that carries part of λgt10 *lacZ*) was inserted into pHM5 |
| pAdHM4 | adenovirus type 5 genome with deletions in E1 and E3 regions, bla | Figure 2. [18] |
| pAdNY56 | *lacZ*^+^, *bla* | I-CeuI-PI-SceI fragment of pNY56 (that carries part of λgt10 *lacZ*) was inserted into pAdHM4. |
| pAdNY57 | *lacZ*^-^, Glu461Gly (GAA to GGA), *bla* | I-CeuI-PI-SceI fragment of pNY57 (that carries part of λgt10 *lacZ*) was inserted into pAdHM4. |
| pAdNY58 | *lacZ*^-^, Tyr105Stop (TAT to TAG), *bla* | Figure 2. I-CeuI-PI-SceI fragment ofpNY58 (that carries part of λgt10 *lacZ*) was inserted into pAdHM4. |
| Adenovirus recombinants | |  |
| AdNY56 | *lacZ*^+^ | PacI fragment of pAdNY56 (that carries the whole recombinant adenovirus genome) was transfected into 293 cells. Released from these cells and purified. |
| AdNY57 | *lacZ*^-^, Glu461Gly (GAA to GGA) | PacI fragment of pAdNY57 (that carries whole recombinant adenovirus genome) was transfected into 293 cells. Released from these cells and purified. |
| AdNY58 | *lacZ*^-^, Tyr105Stop (TAT to TAG) | Figure 2. PacI fragment of pAdNY58 (that carries whole recombinant adenovirus genome) was transfected into 293 cells. Released from these cells and purified. |
